# Laparoscopic Sac Excision as a Definitive Technique for Inguinal Hernia Management in Pediatric Females: A Prospective Study

**DOI:** 10.7759/cureus.52940

**Published:** 2024-01-25

**Authors:** LKR Shanbhogue, Tuqa A Alsinan, Mohammad Samer Baki, Mohammed T Almohaidly, Mohammad Alonazi, Abdullah Y Almusallam

**Affiliations:** 1 Pediatric Surgery, Maternity and Children Hospital, Qassim, SAU; 2 Pediatric Surgery, Prince Sultan Military Medical City, Riyadh, SAU; 3 Public Health, Alfaisal University College of Medicine, Riyadh, SAU; 4 Pediatric Surgery, Ministry of National Guard Hospital, Riyadh, SAU

**Keywords:** pediatric inguinal hernia repair, prospective study, recurrent inguinal hernia, laparoscopic inguinal hernia repair, pediatric suregery

## Abstract

Introduction: The utilization of laparoscopic techniques in the management of inguinal hernias among pediatric patients has seen a rising trend. We aimed to assess the efficacy of laparoscopic excision of the hernial sac as a suitable approach for managing inguinal hernias specifically in female patients and conducted a prospective study to investigate this hypothesis.

Methods: Over a comprehensive four-year period, a total of 99 hernias in 69 female patients were surgically addressed using laparoscopic methods. The surgical procedure primarily involved the laparoscopic inversion and excision of the hernial sac without subsequent distal suturing.

Results: During the initial phase of the study, two cases encountered recurrences within 48 hours post-operation, potentially attributed to incomplete excision. However, in the subsequent period, no further recurrences were recorded.

Conclusion: Our study findings support the contention that laparoscopic excision of the sac, without adjunctive closure of the peritoneum, suffices as an effective approach for managing inguinal hernias in female pediatric patients.

## Introduction

Pediatric surgeons commonly encounter inguinal hernias, with the gold standard for repair involving open techniques encompassing dissection, high ligation, and sac excision. However, the growing trend toward laparoscopic approaches seeks to offer cosmetic benefits and mitigate the risk of contralateral metachronous hernias. Additionally, laparoscopy serves as a diagnostic aid in identifying rare cases such as congenital Androgen Insensitivity Syndrome (AIS) in girls [1]. The incidence of inguinal hernias in children varies from 1% to 5%, influenced by factors like prematurity and associated conditions. Notably, most studies indicate a male-to-female ratio of 1:10 [1].

Laparoscopic techniques fall into two categories: extracorporeal and intracorporeal. Extracorporeal techniques typically involve placing a purse-string suture (absorbable/non-absorbable) around the internal inguinal ring under laparoscopic guidance, followed by subcutaneous tying. Various modifications using different needles and sutures have been proposed [2]. In contrast, intracorporeal techniques encompass sac division with proximal peritoneal closure, distal suturing, and in girls, sac eversion with Endo loop application or complete sac cauterization, termed the burnia technique [3-6]. Observations from laparoscopic orchidopexy indicate that dividing the hernial sac/peritoneum at the internal ring level without attempting peritoneal defect closure does not elevate the risk of subsequent inguinal hernias. This observation, coupled with reports suggesting that high sac division in open techniques does not escalate recurrence rates compared to ligation, proposes that sole laparoscopic sac division might suffice [7]. This notion is particularly intriguing in girls due to the distant location of gonadal vessels from the sac and the absence of a vas deferens. Although some reports exist on laparoscopic sac resection or division, this practice lacks widespread adoption [8-10]. Our hypothesis revolves around the adequacy of laparoscopic excision of the hernial sac alone for managing inguinal hernias in girls, forming the basis for this prospective study.

## Materials and methods

All female patients admitted at the maternity and children hospital in Qassim, Saudi Arabia, with a diagnosis of inguinal hernia, both elective and emergency cases, the study was planned in 2019 to 2023, spanning four years, underwent laparoscopic herniotomy was followed prospectively. Before the procedure, informed consent was diligently obtained from the parents, comprehensively detailing the potential advantages and risks associated with laparoscopy, including a slightly increased likelihood of recurrence compared to open techniques.

Operative procedure

Under general anesthesia and endotracheal intubation, a standardized approach utilizing a three-trocar technique was employed. A 5-mm scope was inserted through the umbilical port, while two 3.5 mm trocars were positioned on either side for instrumentation. Following confirmation of the diagnosis, a thorough inspection of the opposite internal ring was conducted to detect any patent processus. Specific measurements of the internal ring size were not obtained or emphasized during the procedure.

Additionally, inspection of the uterus and ovaries was performed to rule out the possibility of congenital AIS. The hernial sac was meticulously grasped, everted, and subsequently excised at the internal ring level using either a diathermy hook or scissors. In most instances, inclusion of the round ligament of the uterus occurred with the sac. The excised sac was extracted through the trocar. Closure of the 5 mm trocar site involved a single deep suture along with subcuticular skin closure, whereas Steri Strips were applied to the 3.5 mm trocar sites. Patients aged over one year were managed as day cases, while those under one year were discharged the following day.

## Results

A total of 69 female pediatric patients participated in the study, ranging in age from two months to 13 years, with 29 cases aged less than one year. The weight of patients varied from 2 to 42 kg.

**Figure 1:**
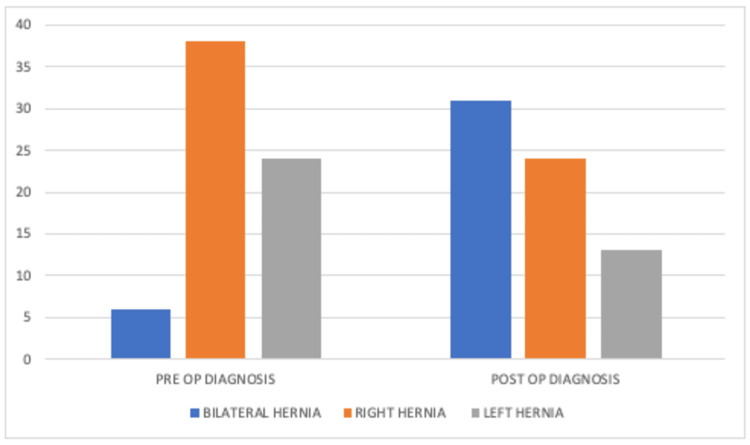
Preoperative diagnosis and actual operation finding in females with inguinal hernia

Preoperatively, the diagnosis revealed bilateral hernias in six cases, right-sided hernias in 39 cases, and left-sided hernias in 24 cases. During laparoscopy, a patent processus was observed on the opposite side in 25 cases initially diagnosed as unilateral hernias (36.7%). All cases with identified patent processus were managed similarly, treating the hernial sac accordingly. These findings are summarized in Table 1 and Figure 1.

**Table 1:** Details of the patients included in the study

Duration of study	From the year 2019 to 2023
Age of our cases	From 2 months to 13 years
Body weight	From 2 to 42 Kg
Total of pateints	69
Pre – op Bilateral Hernia	6
Post – op Bilateral Hernia	31
Pre – op Right Hernia	38
Post – op Right Hernia	24
Pre – op Left Hernia	24
Post – op Left Hernia	13
Total No of operated hernia	99
Conversion to open No	2
Recurrence No	2
Follow up	From 3 months to 3 years

Notably, two cases required conversion from laparoscopic intervention. One patient initially diagnosed with an incarcerated inguinal hernia involving the ovary was found, upon laparoscopy, to have both internal rings closed. Subsequently, an inguinal incision revealed a hydrocele of the canal of Nuck, which was excised. In the other case, bilateral hernial sacs were identified. While the right side was managed laparoscopically, the left side presented an incarcerated ovary in the inguinal region, resistant to laparoscopic reduction despite manipulation. Upon canal exploration, the ovary firmly adhered to the external inguinal ring with multiple adhesions, necessitating adhesiolysis for reduction.

Throughout the study period, a total of 99 hernias were treated laparoscopically, and the follow-up duration ranged from one month to three years. Initially, two early recurrences were observed within 48 hours post-operation during the early phase of the study. However, subsequent to these occurrences by physical examination that were managed conservatively, no further recurrences were reported. Furthermore, no other complications were noted during the follow-up of four to six weeks period.

## Discussion

In the realm of managing inguinal hernias in children, various surgical approaches have been adopted, including single-incision laparoscopic Surgery (SILS), two-trocar, and classical three-trocar techniques. In this study, our preference was for the three-trocar technique due to our team's familiarity, although the primary focus remains on the sac division, as procedural preferences may vary among operators. Cauterization of the peritoneum at the level of the internal ring, known as the “Burnia” technique, has been proposed to reduce recurrence rates. While this technique appears straightforward, concerns arise regarding heat conduction to adjacent structures, such as the fallopian tube and ovary. Thus, if the excision of the sac alone yields equally favorable outcomes, the necessity for cauterization becomes debatable. Alternative methods, like laparoscopic sac inversion and endoloop application, have been described; however, our premise suggests that sac excision suffices, eliminating the need for ligation [6-10].

Percutaneous Internal Ring Suturing (PIRS) techniques, including modifications, have gained popularity for inguinal hernia repair. Studies report varying recurrence rates, with some indicating slightly higher rates compared to open techniques. Our concern with the PIRS technique lies in its dependence on suture integrity, especially during the early postoperative period, as the sac is neither divided nor excised [1,2]. Other studies have attempted to replicate open herniotomy steps laparoscopically with sac division and distal peritoneal suturing, claiming favorable outcomes. However, this technique necessitates significant laparoscopic skills and may be deemed unnecessary in certain scenarios [1-4].

Notably, laparoscopy offers unique advantages such as cosmetic benefits, pelvic organ inspection, and contralateral patent processus vaginalis treatment. Incidence rates of contralateral metachronous hernias have been detailed, emphasizing the higher likelihood of initial left-sided hernias among females [1]. Early recurrences observed in our study were attributed to potential technical faults, possibly due to incomplete sac excision, leading to a residual wedge and subsequent recurrence. However, subsequent cases showed no recurrences. Our technique involves round ligament excision along with the hernial sac, akin to open herniotomy where this practice has been established without reported issues in girls [2-6].

An alternative viewpoint proposes that complete sac excision may not be imperative, suggesting laparoscopic sac transection near the internal ring to create a circumferential defect in the peritoneum, potentially sufficient for repair. Studies indicating comparable outcomes between sac transection and distal ligation warrant further exploration and simplification of laparoscopic herniotomy techniques in both genders [9-15]. While various techniques exist for laparoscopic herniotomy, further research is warranted to refine and simplify approaches, optimizing outcomes for pediatric patients.

It is important to acknowledge several limitations of our study. First, the prospective nature of the study introduces inherent biases, such as surgeon selection bias and incomplete data. Second, our study was conducted at a single center in Saudi Arabia, which may limit the generalizability, sample size, duration of follow-up, standardization of the technique, randomization, blinding, and comparison of other techniques of our findings to other populations. Future multi-center studies with larger sample sizes are needed to validate our findings and further explore more findings and surgical outcomes.

## Conclusions

Our study on laparoscopic sac excision in female pediatric patients with inguinal hernias showed promising outcomes, with no recurrences or complications during the follow-up period. Laparoscopy offers cosmetic benefits, contralateral hernia management, and pelvic organ inspection. Further research is needed to validate and refine this technique for optimal outcomes in pediatric inguinal hernia management.
